# Freezing/thawing pretreatment of dormant *Aspergillus niger* spores to increase the Cr(vi) adsorption capacity: process and mechanism

**DOI:** 10.1039/d0ra10198b

**Published:** 2021-02-17

**Authors:** Binqiao Ren, Luyang Zhao, Yanli Wang, Xiaoxiao Song, Yu Jin, Fengju Ouyang, Chongwei Cui, Hongwei Zhang

**Affiliations:** School of Environment, Harbin Institute of Technology Harbin 150090 People's Republic of China cuichongwei1991@126.com +86 18845181188; Institute of Advanced Technology, Heilongjiang Academy of Sciences Harbin 150080 People's Republic of China hljdezx@126.com +86 13359992319

## Abstract

Hexavalent chromium is a widespread pollutant that threatens ecological and human health. However, its removal from the environment is limited by the high cost and energy consumption rate of current technologies. In this study, the Cr(vi) biosorption mechanism of *Aspergillus niger* spores pretreated by freezing/thawing was studied by batch experiments and surface chemistry analyses. The results indicated that pretreatment enhanced the spores' Cr(vi) removal efficiency. The cell surface, internal functional groups, and morphology of the freezing/thawing-pretreated spores (FTPS) before and after Cr(vi) loading were characterized by advanced spectroscopy techniques such as SEM-EDAX, XPS, FTIR, and FETEM analyses. The SEM and BET data showed that the surface of FTPS was rougher than that of untreated spores. The XPS data showed that FTPS bio-transformed Cr(vi) into Cr(iii). The intracellular localization of chromium was visualized by FETEM, and both surface and intracellular structures removed Cr(vi) following pseudo-second-order biosorption kinetics. The biosorption dynamics of Cr(vi) fit the Langmuir isotherm model describing a monolayer. These results suggest that freezing/thawing pretreatment of *A. niger* spores could lead to the development of a novel, efficient biomaterial for the removal of Cr(vi).

## Abstract 2

Freezing/thawing pretreatment of *A. niger* spores could lead to the development of a novel, efficient biomaterial for the removal of Cr(vi).

## Graphical abstract

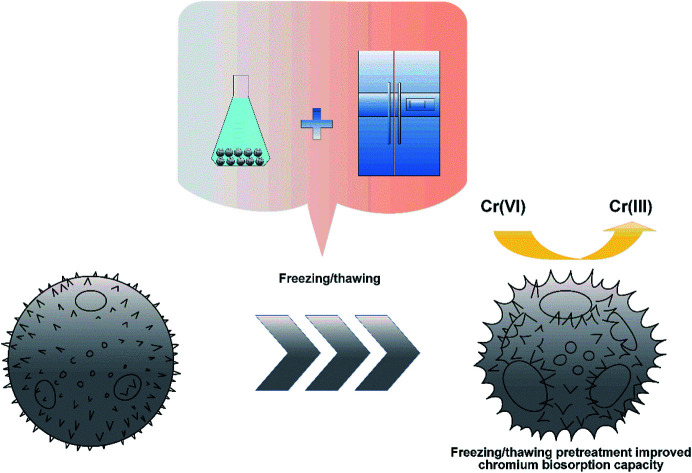

## Introduction

Hexavalent chromium is widely used in electroplating, textile dyeing, stainless steel, leather, wood treatment, and other industries. However, Cr(vi) has been identified as one of 17 chemicals that pose high risks to human health by the United States Environmental Protection Agency (USEPA).^1^ In particular, the release of chromium from anthropogenic sources to natural freshwaters must be considered with care.^2^ Exposures to Cr(vi) can severely impact the health of flora and fauna, even at low concentrations (10 mg L^−1^).^3^

The USEPA's limit for the concentration of Cr(vi) in water was set at 0.05 mg L^−1^, while the concentration of total Cr (including Cr(iii), Cr(vi), and other forms) was set at 2 mg L^−1^.^4^ In response to regulatory guidelines, some researchers have been working to develop effective techniques for the removal of Cr(vi) from wastewaters or the conversion of it into less toxic forms such as Cr(iii). The removal of heavy metal ions from water is usually accomplished through chemical precipitation reactions. Physical and chemical treatments are easy to implement and can aid in metal recovery, but when there is a low concentration of metal ions in the waste stream, these metals can be difficult to remove.^5,6^ Thus, most of these techniques only work well at high metal concentrations, and their efficiency is reduced by the presence of other interfering metals.^2^ These shortcomings need to be considered in order to develop a stable and economical method of metal removal.

The biosorption method is suitable for the treatment of low concentration metal solutions,^1^ in which biosorption refers to the ability of certain types of microorganisms to biologically complex and accumulate pollutants in aqueous solutions.^7^ Similar to chemical adsorption processes, the amount of contaminants attached to the adsorbent depends on the available functional groups and structures of the cell surface. In general, the biosorption process is largely influenced by pH.^8^ Bacteria, yeast, algae, plants, and fungi have been shown to possess a metal adsorption capacity;^9^ however, fungi often exhibit better heavy metal biosorption performances than other taxa. Biosorption methods have several advantages including low operational costs, low energy requirements, no secondary pollution, and the possibility of reuse of recovered metals.^10^ Presently, adsorptive materials have been developed from various fungal strains, and their Cr removal efficiencies have been analyzed. To improve the biosorption capacity, many researchers have studied physically and chemically modified species, and *Aspergillus flavus* biomaterials infused with Fe^2+^ ions were found to have enhanced Cr removal performances and improved stickiness of the biomaterial.^11^ Ramrakhiani *et al.* developed physically and chemically modified fungi, and the results indicated that acid pretreatment can effectively increase the biosorption efficiency, while alkali conditions reduced it.^12^ Three main chemical modifications have been assessed, namely, carboxyl esterification, base hydrolysis, and amino methylation, and the amino methylation-modified fungal biosorbent was found to be the best.^13^ Physical modifications of fungi are easier to implement than chemical modifications and will produce less secondary pollution. However, few studies have been conducted on the mechanism of the biosorption of Cr(vi) on freezing/thawing-pretreated spores of filamentous fungi.

In this study, fungal spores were pretreated by freezing/thawing to (1) evaluate the biosorption capacity under different freezing temperatures and freezing/thawing times, (2) investigate the influence of four operational factors (pH, contact time, initial concentration, and temperature) on the biosorption capacity and kinetics of fungal spores pretreated with freezing/thawing, and (3) explore the biosorption mechanism of freezing/thawing-pretreated fungal spores.

## Experimental

### Biomass preparation

*Aspergillus niger* spores were produced as a reproductive propagule in solid media during the late growth stage.^14^

#### Preparation of *A. niger* spores

*Aspergillus niger* CMCC98003 was acquired from the National Center for Medical Culture Collections (CMCC). The spore concentration of the final suspension was adjusted to 10^*n*^ (10^8^–10^9^) CFU mL^−1^ with distilled water and determined by using a hemocytometer. The *A. niger* spores fermented on solid medium (potato dextrose agar, PDA) were collected and stored at room temperature (298–303 K) for subsequent adsorption experiments.^15^

#### Freezing/thawing pretreatment of *A. niger* spores

Spores of *A. niger* (0.1 g) were suspended in pH 2 distilled water (20 mL). Nine samples were prepared—four samples were held under 193 K for one to four cycles and four samples were held under 253 K for one to four cycles for 6 h, and the untreated spores served as the control. The spores treated with several freezing/thawing cycles and under different freezing temperatures were then compared. Then, 100 mL K_2_Cr_2_O_7_ (100 mg L^−1^) solution and 0.1 g biomass were placed in 250 mL conical flasks. The conical flasks were shaken at 303 K (150 rpm) for 4 h. After biosorption, the biomass was separated from the liquid with a filter paper (Whatman 45), and the remaining Cr(vi) ions in the solution were analyzed.

### Chromium analysis

Colorimetry is used to measure the concentration of hexavalent chromium. In acidic solution, 1,5-diphenylurea reacts with Cr(vi) to form a pink complex, which can be determined by spectrophotometry at 540 nm.

A total of 2.828 g K_2_Cr_2_O_7_ was dissolved in 1 L of distilled water to make the Cr(vi) stock solution (1000 mg L^−1^). The stock was then diluted to the required concentration (which ranged from 25 to 200 mg L^−1^).^12^ When *q* (mg g^−1^) is balanced, the amount of Cr(vi) adsorbed by untreated *A. niger* spores (US) or freezing/thawing-pretreated spores (FTPS) represents the amount of metal absorption, which can be calculated as follows:1
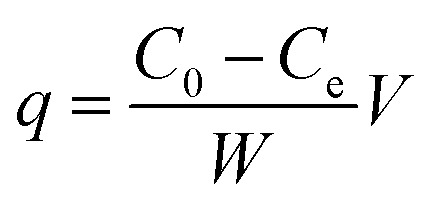
Here, *C*_0_ and *C*_e_ are the initial and equilibrium concentrations of Cr(vi) in the solution (mg L^−1^), respectively, *V* is the solution volume, and W is the dry weight of the biomass (US and FTPS) (g).

### Biosorption experiments

The biosorption capacity of Cr(vi) for US and FTPS was obtained by regularly measuring the Cr(vi) concentrations in batch processing tests. The biosorption experiments were carried out as follows. A total of 1 g L^−1^ biomass adsorbent was added into a 250 mL conical flask containing 100 mL of a 100 mg L^−1^ chromium(vi) solution. The reaction conditions were as follows: initial pH value, 2.0; temperature, 303 K; rpm, 120; duration, 4 h. The concentration of Cr(vi) was then determined using ultraviolet spectrophotometry.^16^ Because the absorption of Cr(vi) by US was significantly lower than that of FTPS biomass (193 K per cycle), we used the latter material (FTPS) for further adsorption studies.

The effects of pH (2–7), contact time (15–300 min), initial concentration (25–250 mg L^−1^), and temperature (293–313 K) on the biosorption of Cr(vi) by FTPS biomass were studied.

#### Effect of the solution pH

The solution pH ranged from 2 to 7, with increments of 1, and the pH was maintained by using a Lewis acid and base (HCl or NaOH). The biosorption experiments were conducted at a Cr(vi) concentration of 100 mg L^−1^ and with 0.1 g of FTPS or US in 100 mL of the solution.

#### Effect of the contact time

The contact time ranged from 25–300 min in the adsorption capacity experiments. The US and FTPS (0.1 g) were added to a 100 mL solution containing Cr(vi) at 100 mg L^−1^, with a pH of 2.0 and temperature of 303 K.

#### Effect of the initial solution concentration

The initial concentration ranged from 25–250 mg L^−1^ in the adsorption capacity experiments. The US and FTPS (0.1 g) were added to a 100 mL solution containing Cr(vi) at 100 mg L^−1^, with a pH of 2.0 and temperature of 303 K. These experiments were conducted for 4 h.

#### Effect of the temperature

The temperatures of 293 K, 303 K, and 313 K were investigated in the adsorption experiments. The US and FTPS (0.1 g) were added to a 100 mL solution containing Cr(vi) at 100 mg L^−1^, with a pH of 2.0 and temperature of 303 K. The experiments were conducted for 4 h.

### Isotherms

The isotherms were obtained to calculate the Cr(vi) adsorption capacities of the FTPS. The experimental data were applied separately to Langmuir and Freundlich models. The Freundlich equation is given below (heterogeneous):

2 ln(*q*_eq_) = *n* ln(*c*_eq_) + ln *K*_F_

Here, *q*_eq_ is the metal biosorption capacity at equilibrium (mg g^−1^), *c*_eq_ is the metal ion concentration at equilibrium (mg L^−1^), and *K*_F_ and *n* are the Freundlich constants.

The linearized Langmuir equation is given below (monolayer):3
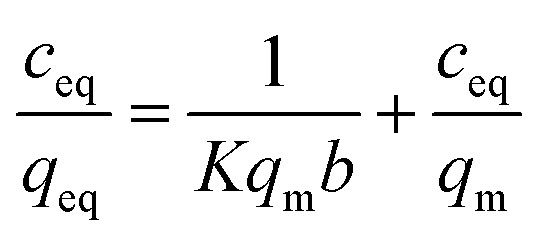
Here, *q*_eq_ is the metal biosorption capacity at equilibrium (mg g^−1^), *c*_eq_ is the metal ion concentration at equilibrium (mg L^−1^), *q*_m_ is the monolayer capacity (mg g^−1^), and *b* is the Langmuir constant (L mg^−1^).

### Kinetics

The kinetics of the biosorption of Cr(vi) on the FTPS were investigated. The metal concentrations were measured at 5, 15, 30, 60, 90, 120, 180, 240, and 300 min. To determine the metal content, the suspensions were filtered and analyzed by using atomic absorption spectroscopy (AAS). The rate of adsorption was evaluated by using the integrated forms of pseudo-first-order and pseudo-second-order reaction equations as described below:

First-order:

4 ln(ln *q*_eq_ − *q*_*t*_) = ln(*q*_eq_) − *k*_1_*t*

Second-order:5
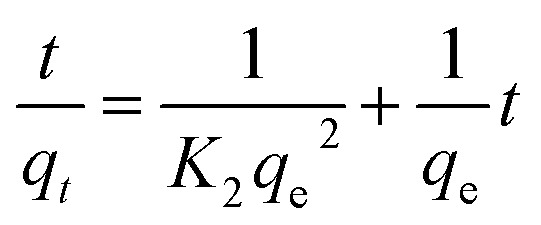
Here, *q*_eq_ is the metal biosorption capacity at equilibrium (mg g^−1^), *q*_*t*_ is the metal biosorption capacity (mg g^−1^) after time *t* (min), *k*_1_ is the first-order rate constant (min^−1^), and *k*_2_ is the second-order rate constant (g mg^−1^ min^−1^).

### Spore characterization

Scanning electron microscopy and energy-dispersive X-ray spectroscopy (SEM-EDAX) were used to observe the surface morphology of biosorption materials before and after biosorption, and the heavy metal ions adsorbed by the biosorption materials were evaluated. For these analyses, the FTPS were applied to a copper conductive paste to obtain a better image contrast than that achieved by spraying gold. This image was viewed by using a Japanese SU8010 scanning electron microscope (SEM). The electron density analysis of different elements on the material (*i.e.*, C, N, O, Cr) was carried out on an American Tales F200x field emission transmission electron microscope (FETEM). Zeta potential of US and FTPS before and after biosorption were measured to analyze the biosorption mechanism. Then, X-ray photoelectron spectroscopy (XPS) was used to analyze the valence state of the adsorbed metal ions in the biosorption materials and to judge whether the valence state of metal ions changed during the adsorption process. The equipment model used was a PHI5400 ESCA. Agilent Fourier transform infrared (FTIR) technology was used to analyze the changes of functional groups before and after US and FTPS biosorption.

## Results and discussion

### Effect of the pretreatment temperature and number of cycles

**Fig. 1:**
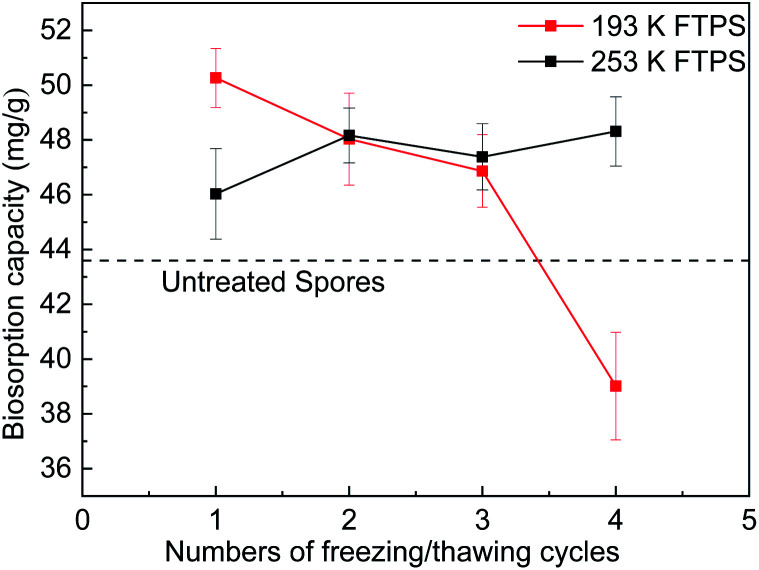
Biosorption capacity of FTPS treated at different freezing/thawing temperatures and for different numbers of cycles.

The temperature and number of freezing/thawing cycles are important parameters, as the changes in them affect the surface and structure of biomass as well as protein formation. To identify the conditions that would achieve the maximum removal efficiency, experiments were conducted at temperatures of 193 and 253 K and one to four cycles (Fig. 1), with the US as a control. The biosorption capacities of the FTPS held under 193 K were 50.2, 48.0, 46.8, and 39.0 mg g^−1^, respectively, as the number of cycles increased from one to four, while those held under 253 K were 46.0, 48.2, 47.4, and 48.3 mg g^−1^, respectively. Therefore, the FTPS demonstrated a better biosorption capacity than the US (43.6 mg g^−1^).^17^ The optimum Cr(vi) biosorption capacity was achieved under a freezing/thawing temperature of 193 K with one cycle. The increase in the capacity of the FTPS could have been related to the collapse of the cell walls and membranes by the freezing/thawing pretreatment, which allowed more Cr(vi) to enter inside the spores. However, the biosorption capacity then decreased as the number of freezing/thawing cycles under the temperature of 193 K increased. At 193 K, after four cycles, the biosorption capacity decrease for FTPS could have been related to the decomposition of the spores. The biosorption capacity sharply decreased here. Comparing with the condition of 193 K, the condition of freezing-thawing pretreatment at 253 K is milder. It does not cause great changes in cell structure; the adsorption performance does not decline significantly.

### Effect of the influencing parameters on Cr(vi) biosorption

#### Effect of the pH

**Fig. 2:**
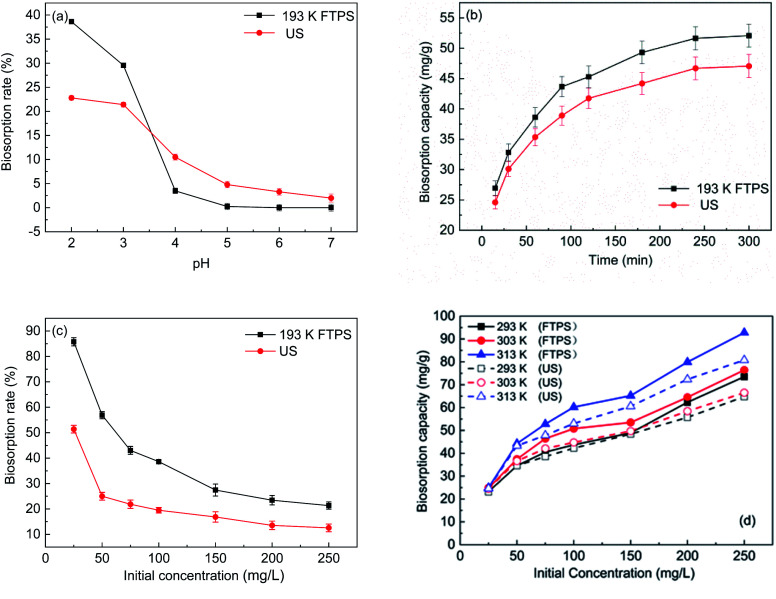
Effect of the pH (a), initial Cr(vi) concentrations (b), time (c), and temperature (d) on Cr(vi) biosorption.

The pH is the main parameter that influences heavy metal removal.^4^ Notably, variations in pH can affect the characteristics of metal ions and the surface charge of biosorbents.^18^ As shown in Fig. 2(a), the Cr(vi) biosorption capacity of FTPS increased with a decrease in the pH, and the maximum value was reached at pH 2. The biosorption rate of FTPS was higher than that of US. The biosorption rate of FTPS reached to almost 40%, while that of US reached to 23%. At the same time, it was also observed that when the pH range was 4–7, the adsorption capacity of FTPS was lower than that of US. The reason may be that the structure of *Aspergillus niger* spores is stable when the pH is 2–3. As the pH increases, the spores will decolorize. Therefore, we believe that the increase in pH will affect the stability of the spore structure of *Aspergillus niger*, and there will be many holes on the surface of the FTPS after pretreatment. As the pH value increases, the structure of FTPS is more easily destroyed. Therefore, when the pH is greater than 3, the adsorption capacity drops rapidly the adsorption capacity drops more rapidly compared with US. The biosorption rate of both types of spores then decreased as the pH increased. Similar results were observed in previous studies, in which the biosorption capacity of Cr(vi) increased with decreasing pH 7. The decrease in the pH could have increased the protonation of the surface of the spores, thereby resulting in stronger attractions to chromate anions in the solution, with Cr_2_O_7_^2−^, CrO_4_^2−^, Cr_4_O_13_^2−^, and HCrO_4_^−^ as dominant species.^19^

With the increase in the pH value, the concentration of OH^−^ ions increased, and the surface charge of US and FTPS became negative. Chromate ions also have a negative charge in water, and the effect of electrostatic repulsion can reduce the adsorption capacity; hence, with the increase in the pH value, the adsorption capacity decreased sharply. Therefore, the findings demonstrate that the adsorption of chromium on US and FTPS depends on the pH value of the solution, and static electricity plays a major role in this biosorption process.

Zeta potential of US and FTPS before and after biosorption was measured in chromium solution with 100 mg L^−1^, pH of 2 (Table 1). Before adsorption, zeta potential of FTPS was significantly higher than that of US, with the prolongation of reaction time, zeta potential of both US and FTPS decreased. Therefore, the increase of Zeta potential is beneficial to improve the adsorption performance of biosorption materials. The results show that electrostatic adsorption plays an important role in the adsorption process. Similar results were achieved for both the FTPS and US. Therefore, increasing zeta potential is beneficial to the adsorption efficiency, and the experimental results are consistent with the above results of the concentrations and pH profiles.

**Table 1:** Zeta potential data of both US and FTPS samples before and after biosorption studies

Time (min)	US (mV)	FTPS (mV)
0 min	−0.42067	5.17667
240 min	−1.11423	1.62333

#### Effect of the contact time

Fig. 2(b) shows that the Cr(vi) biosorption of the US and FTPS both occurred in two phases, namely, a rapid phase and a slow phase. Previous studies on biosorption also produced similar results for different types of biomass.^15,18^ The rapid phase was sustained for approximately 100 min, during which almost half of the Cr(vi) was absorbed. Additionally, the biosorption capacity and efficiency of FTPS were higher than those of US. The adsorption mechanism of FTPS may involve electrostatic interactions, chelation of functional groups on the cell surface, and biotransformation reactions. After 100 min, the biosorption process became relatively slow, and US also showed a similar trend. Up to 300 min, the slow biosorption process may be due to the large number of occupied biosurfactant adsorption sites.^20^

#### Effect of the initial concentration

The initial concentration also affected the biosorption of Cr(vi) (Fig. 2(c)). The Cr(vi) biosorption rate declined as the initial concentration increased. When the biomass dosage was fixed, the biosorption efficiency decreased dramatically with the increase in the initial concentration. The biomass reactive sites were occupied by the metal ions, and there were no extra surface functional groups to absorb the increasing ions. Furthermore, as the concentration increased, the distance between the metal ions lessened. Owing to static electricity reactions, it became hard to remove the heavy metals with the increasing initial concentrations.^11^

#### Effect of the temperature

Previous studies have demonstrated that the biosorption capacity strongly depends on temperature.^14,15^ Therefore, batch comparison experiments with US were conducted to investigate the biosorption capacity of FTPS at different temperatures (293, 303, and 313 K).

Fig. 1(d) shows the biosorption capacity of FTPS held under different temperatures. As the temperature increased, the biosorption capacity of the treated spores increased markedly. Similar results have been observed for US,^17,18^ and this effect is mainly due to some type of biochemical mechanism in which the adsorption process is promoted by increases in temperature. The Cr(vi) uptake capacity reached 92.8 mg g^−1^ at a temperature of 313 K with an initial concentration of 250 mg L^−1^.

### Biosorption kinetics analysis

**Fig. 3:**
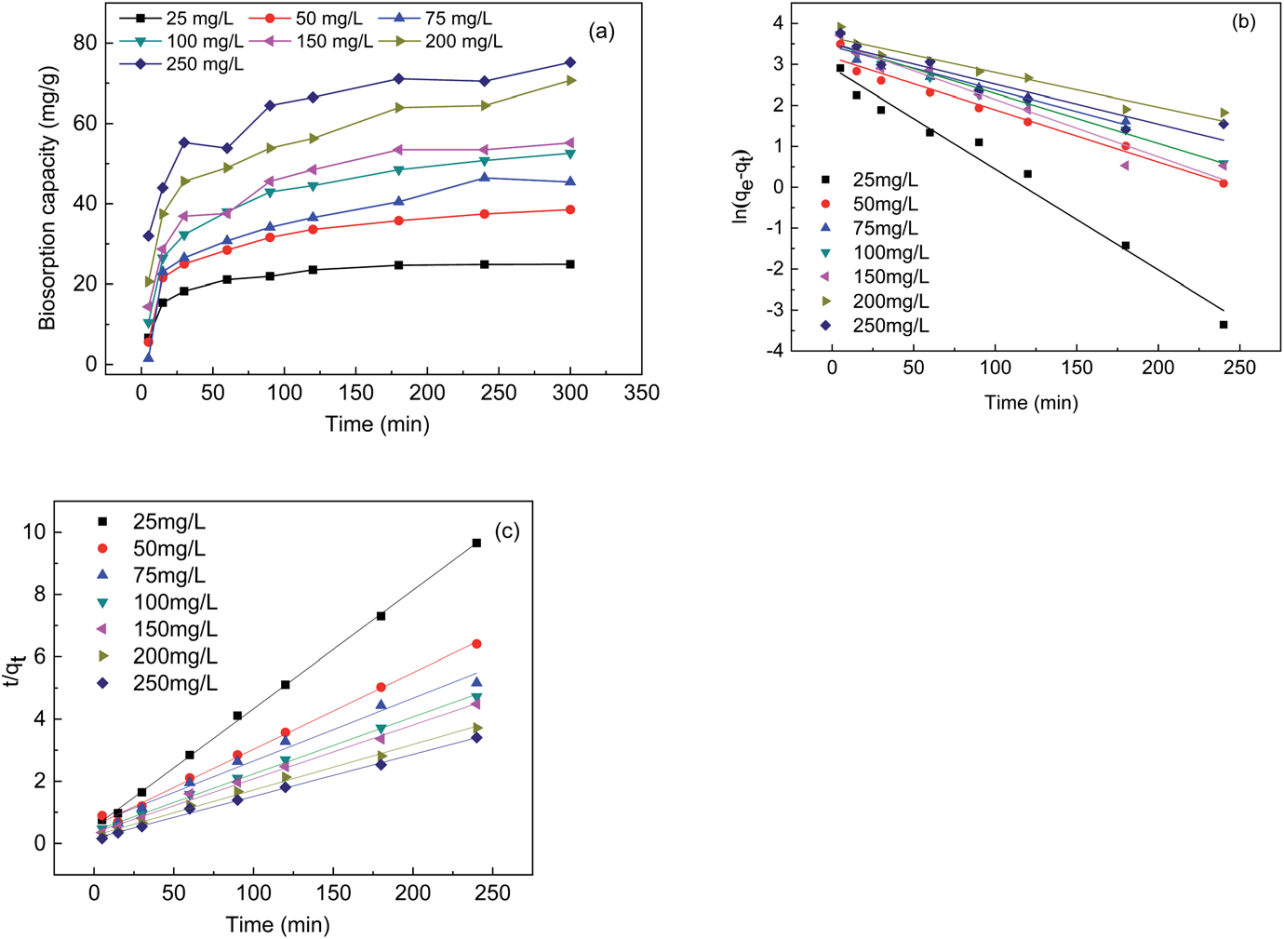
Biosorption capacity under different initial Cr(vi) concentrations (a), the pseudo-first-order model (b), and the pseudo-second-order model (c).

Fig. 3(a) shows the biosorption curves for the changes in the biosorption capacity of the FTPS under different initial Cr(vi) concentrations. The biosorption capacity of the treated spores increased rapidly before 25 min, and the Cr(vi) sorption equilibrium was reached after 100 min. It took 200 min for the US to reach the sorption equilibrium, and even longer times were noted in earlier work.^17^ This indicates that the biosorption rate of FTPS was much higher than that of US. This attribute is critical for the applicability of the biosorbent in real industrial water treatment operations. The kinetics curves (Fig. 3(b) and (c)) were evaluated with the pseudo-first-order and pseudo-second-order reaction models (eqn (4) and (5)), and the parameters that were calculated from these equations are presented in Table 2. The *R*^2^ values of the models showed that the Cr(vi) adsorption of the FTPS fit best with pseudo-second-order model.^3,21^

**Table 2:** Kinetics parameters of the Cr(vi) biosorption rate for FTPS

Initial concentration (mg L^−1^)	Pseudo-first-order			Pseudo-second-order		
	*k*_1_ (L min^−1^)	*q*_cal_ (mg g^−1^)	*R* ^2^	*k*_2_ (g mg^−1^ min^−1^)	*q*_cal_ (mg g^−1^)	*R* ^2^
25	0.02128	24.90625	0.975976	0.0027	26.29	0.99917
50	0.01101	39.06866	0.965404	0.0011	40.63	0.995
75	0.00749	46.79769	0.901246	0.0002	49.48	0.97706
100	0.01095	53.46678	0.976542	0.0008	54.50	0.99749
150	0.01153	55.72111	0.942097	0.0009	57.70	0.99377
200	0.00956	73.72894	0.929089	0.0009	68.12	0.99399
250	0.0114	76.95855	0.881584	0.0011	73.96	0.99597

### Adsorption isotherms

**Fig. 4:**
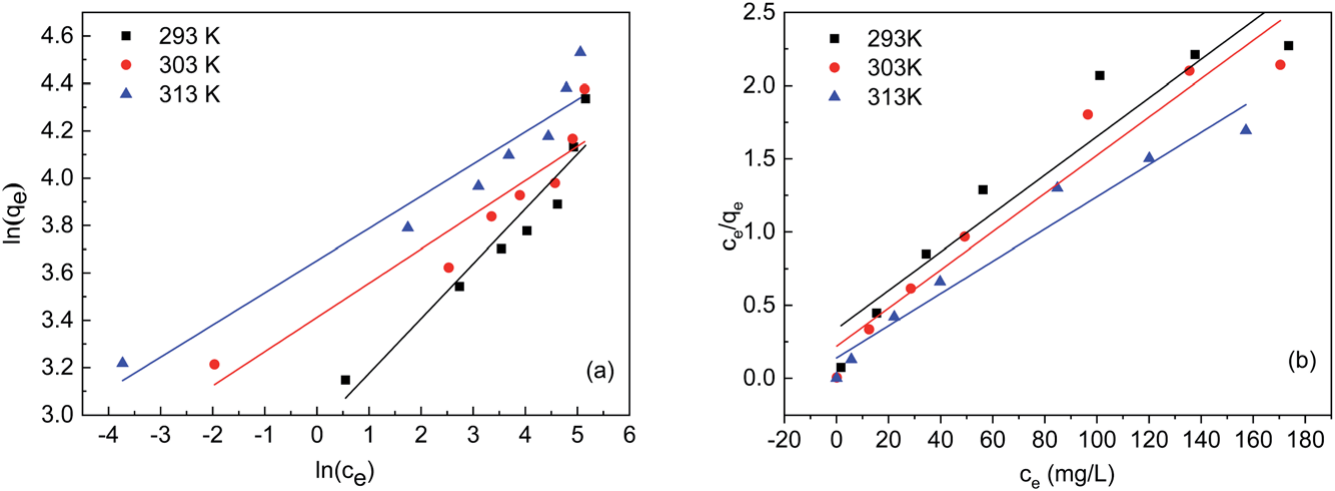
Simulation of the Freundlich (a) and Langmuir (b) isotherms.

Fig. 4 shows the isotherm of FTPS during Cr(vi) biosorption. Two classical models are routinely applied to evaluate the biosorption of chromium ions from aqueous solutions, *i.e.*, Freundlich's and Langmuir's, and both were used here (Fig. 4(a) and (b)). The parameters derived from eqn (2) and (3) are listed in Table 3. The Langmuir model demonstrated a better fit than the Freundlich model, which indicates that the biosorption of the FTPS was a monolayer adsorption process.^22,23^ The biosorption of Cr(vi) on fungal biomass following the Langmuir isotherm has been observed in a previous study.^24^

**Table 3:** Simulation of the isotherm sorption models and corresponding parameters

Temperature (K)	*q*_e,exp_ (mg g^−1^)	Langmuir			Freundlich		
		*q*_max_ (mg g^−1^)	*b* (L mg^−1^)	*R*_1_^2^	*K*_F_ (L mg^−1^)	*n*	*R*_2_^2^
293	76.4	75.98	0.03898	0.8938	18.979	4.3138	0.8975
303	79.6	76.56	0.05969	0.9294	30.322	6.9165	0.8637
413	92.8	90.74	0.07929	0.9495	38.555	7.351	0.9162

### Biomass characterization

#### SEM and EDAX results for US and FTPS

**Fig. 5:**
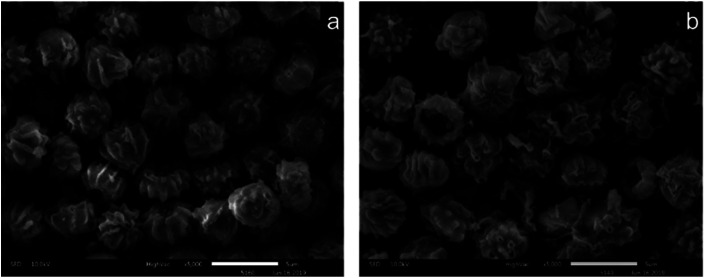
SEM images of the US (a) and FTPS (b).

**Fig. 6:**
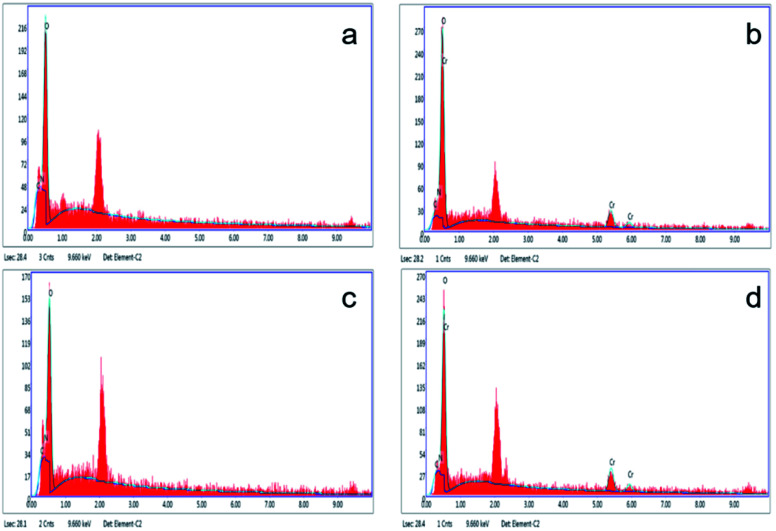
EDAX of results for US obtained before (a) and after (b) Cr(vi) adsorption, FTPS before (c) and after (d) Cr(vi) adsorption.

The freezing/thawing pretreatment could have changed the surface characteristics including physical or chemical features. From the Fig. 5, we can observe that pretreatment may cause surface changes, and the size of FTPS (Fig. 5(b)) seems to be larger than US (Fig. 5(a)). Here, the observed structure of FTPS (Fig. 5(b)) became rougher than that of US (Fig. 5(a)), with the emergence of some holes and wrinkles, increased the specific surface area, tunnels and thereby contributed to the retention of more metal ions; this in turn contributed to the improved biosorption capacity. The deposition of Cr(vi) on US and FTPS was further demonstrated by EDAX (Fig. 6(a)–(d)). The additional peak present in the Cr(vi)-adsorbed biomass showed that the US and FTPS could adsorb Cr(vi).

#### XPS analysis

**Fig. 7:**
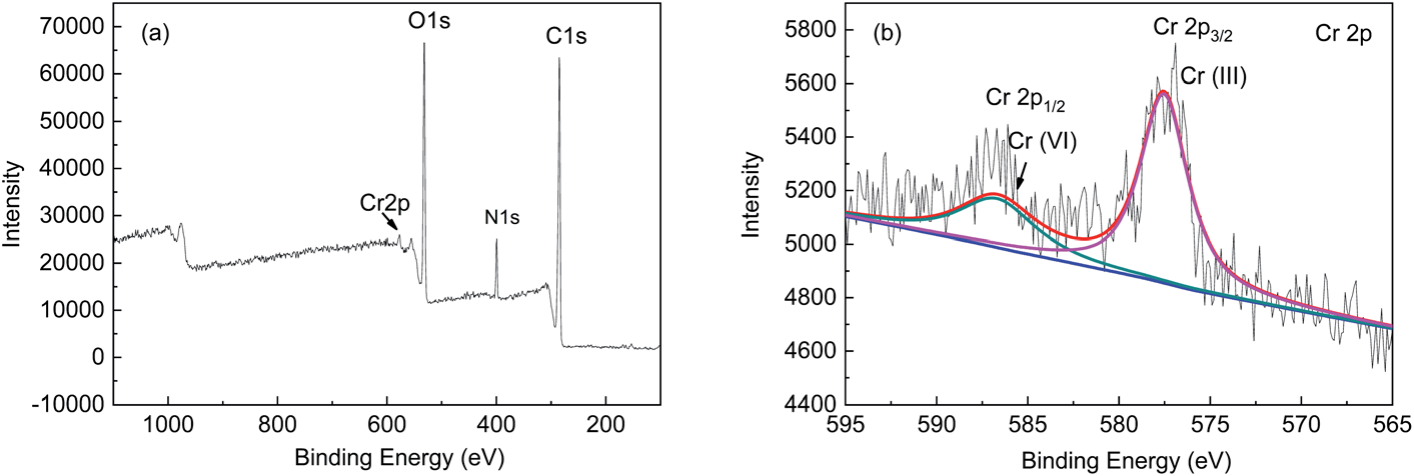
XPS spectra of the FTPS-loaded Cr(vi) (a) and Cr 2p_1/2_ and 2p_2/3_ orbitals (b).

X-ray photoelectron spectroscopy was used to determine the valence state of Cr on the surface of FTPS. Peaks of C, N, O, and Cr can be seen in the low-resolution spectrum (Fig. 7(a)). The high-resolution Cr 2p spectrum of FTPS is shown in Fig. 7(b). The peaks of 576.6–577.9 eV and 586.0–588.0 eV correspond to the characteristic peak binding energies of heavy metals Cr(iii) and Cr(vi) and spore surface functional groups, respectively. The binding energy peaks of the Cr 2p spectrum were 577.7 eV and 586.8 eV, respectively, thus indicating that metal ions in two valence states of Cr(iii) and Cr(vi) existed on the FTPS cell surface. Meanwhile, Cr(iii), which was not initially present in solution, and Cr(vi) adsorbed on FTPS was partially bio-transformed to Cr(iii), when brought into contact with the fungal biomass. In order to better explain the valence transformation of chromium on biomass surface, the pH change of solution before and after adsorption was studied. The pH value of the solution increased from 2.11 to 2.50 after 4 h contact time. The loss of protons in solution may be related to the reduction of Cr(vi). Gardea Torresdey reported that Cr(vi) could be easily reduced to Cr(iii) by positively charged functional groups of biomass,^25^ Park also reported that Cr(vi) was completely reduced to Cr(iii) by contact with the biomass below pH 5.^26^ This study reached the same conclusion as the reported studies, that most of the Cr(vi) on the fungal biomass can transform into the Cr(iii) state.^27^

#### FTIR analysis

**Fig. 8:**
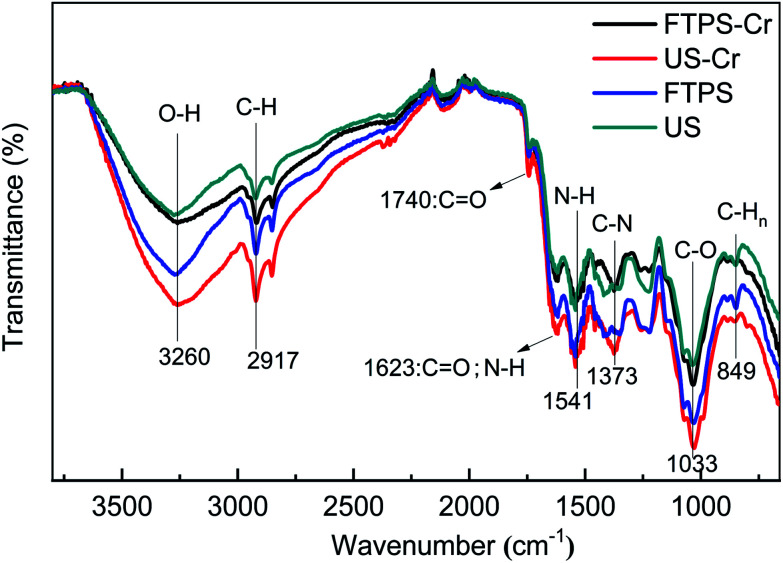
FTIR spectra of the US and FTPS before and after biosorption with Cr(vi).

Fourier transform infrared spectra can provide valuable information regarding the surface functional groups of a material. Fig. 8 presents the FTIR spectra of FTPS and US before and after the adsorption of Cr(vi), in which the spores exhibit a variety of stretching frequencies indicative of the complex nature of the adsorbent. The spectrum of the FTPS exhibited bands at 3268, 2920, 1742, 1625, and 1542 cm^−1^ can be assigned to the alcoholic –OH, carboxylic acid –CH, COOH, –N–H, and amine –C–N stretching. No new peaks appeared comparing with US. The freezing-thawing pretreatment did not cause the changes in functional groups. And the peaks before and after adsorption were similar (FTPS, US, FTPS-Cr, and US-Cr). There was no obvious red shift or blue shift in the infrared curve before and after adsorption, and no new peak appeared, which proved that the surface functional groups of biosorption materials did not participate in the adsorption process of hexavalent chromium. Therefore, electrostatic adsorption may play a major role in the adsorption process.

#### FETEM analysis

The FETEM analysis diagram shows the composition and distribution of elements in FTPS and US.

**Fig. 9:**
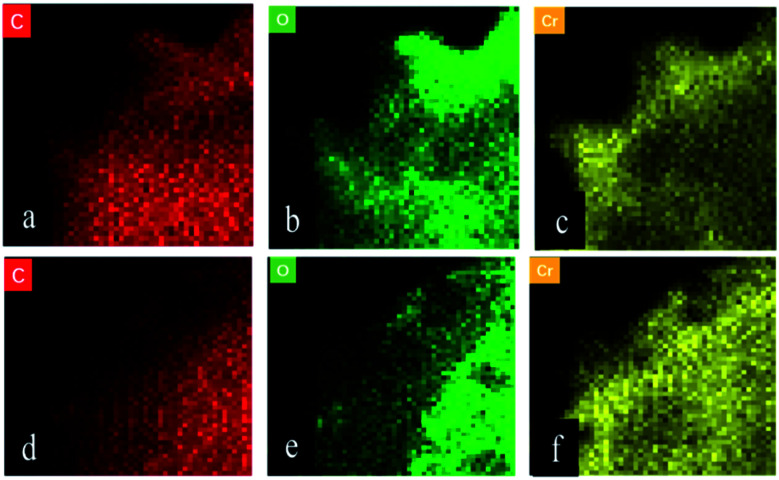
FETEM images of the Cr(vi)-loaded US (a–c) and FTPS (d–f).

Fig. 9(a), (b), and (c) respectively show the presence of C, O, and Cr on US after biosorption, and the results indicate that the biomass can adsorb Cr(vi). Fig. 9(d), (e), and (f) show the presence of C, O, and Cr on FTPS after biosorption. Here, Cr exists not only on the surface, but also inside FTPS, which may explain its high biosorption capacity. Additionally, the elemental density of C and N was lower than that of the US, which might have been due to the leakage of the intracellular structures of the spores after freezing/thawing pretreatment. The FTPS exhibited more wrinkles and cracks on their surfaces; therefore, Cr(vi) diffused to the inside of FTPS from the cracks, thereby reducing the abundance of internal proteins and polysaccharides.

### Removal mechanism by the FTPS biomass

The studies on the influencing factors, kinetics, and adsorption isotherm of the biosorption process showed that the biosorption rate at pH 2.0 was faster than that at pH 3.0, 4.0, 5.0, and 6.0, and the biosorption efficiency was similar with the increase in the contact time. The adsorption occurred in accordance with the Langmuir isotherm model, which indicates that the surface was a monolayer. Therefore, the overall adsorption of Cr(vi) by FTPS biomass is complex, and the main adsorption mechanisms include electrostatic attractions, and biological conversion. From the adsorption kinetics of FTPS on Cr(vi), it can be seen that the adsorption process includes a rapid reaction stage and slow reaction stage. The early rapid reaction was due to the large number of surface-active points on the adsorbent. With the extension of the reaction time, the active adsorption points neared saturation and the reaction speed decreased. The lower pH value resulted in a higher adsorption rate, thus proving that the active site of protonation binds to CrO_4_^2−^ or Cr_2_O_7_^2−^ ions under electrostatic action. This suggests that electrostatic attraction is the main driver of absorption in FTPS.^25^ The slow stage of biosorption may be related to the diffusion of ions in cells, in which the reductive groups in FTPS partially reduce Cr(vi) to Cr(iii) (analyzed through XPS analyses), and the surface and internal structure of FTPS are the main factors. The carbonyl and hydroxyl groups of FTPS (analyzed through FTIR) may be electron donors in the reduction reactions. The structure of US was obviously changed by the freezing/thawing pretreatment. The surface of the FTPS emerged with some holes and wrinkles, which might have increased the specific surface area and tunnels. Freezing/thawing pretreatment of *A. niger* spores resulted in higher adsorption of Cr(vi) than that with US. This could be attributed to the biosorption not only on the surface, but also on the inside of spores (analyzed through FETEM) owing to the characteristic structure of the FTPS.

## Conclusion

In this study, the biosorption of Cr(vi) ions from an aqueous solution using FTPS was successfully realized.

(1) The adsorption characteristics of FTPS and US under different pH levels and different biological stress doses were compared. Under the same reaction conditions (a biosorbent dosage of 0.1 g, pH of 2, 100 rpm, reaction time of 2 h), the biosorption capacity of FTPS (mg g^−1^) was significantly higher than that of US, and the pretreatment resulted in an increase of almost 16%. Thus, FTPS can be used as an effective absorbent to quickly remove Cr. This is a critical factor for the applicability of the biosorbent in real wastewater treatment applications.

(2) The Cr(vi) adsorption mechanism of FTPS was investigated through FETEM analyses. Unlike with US, biosorption did not only occur on the surface. The freezing/thawing pretreatment destroyed the cell membrane, thereby allowing the Cr(vi) to penetrate the spores and bind elsewhere. This also led to the leakage of some intracellular compounds.

(3) The adsorption of Cr(vi) onto these synthesized biosorbents followed the Langmuir isothermal and pseudo-second-order kinetic models, thus indicating that chemical adsorption was the dominant mechanism.

(4) Even a freezing/thawing pretreatment under 193 K can improve the biosorption capacity. Therefore, freezing/thawing provides a novel method to pretreat fungi. However, one limitation is the high cost of the process. Future studies should explore more economical pretreatment technologies.

## Conflicts of interest

There are no conflicts to declare.

## Supplementary Material
